# Enzymatic Synthesis of Sorboyl-Polydatin Prodrug in Biomass-Derived 2-Methyltetrahydrofuran and Antiradical Activity of the Unsaturated Acylated Derivatives

**DOI:** 10.1155/2016/4357052

**Published:** 2016-09-07

**Authors:** Zhaoyu Wang, Yanhong Bi, Rongling Yang, Xiangjie Zhao, Ling Jiang, Chun Zhu, Yuping Zhao, Jianbo Jia

**Affiliations:** ^1^School of Life Science and Food Engineering, Huaiyin Institute of Technology, Huai'an 223003, China; ^2^College of Food Science and Light Industry, Nanjing Tech University, Nanjing 211816, China

## Abstract

Efficient and highly regioselective synthesis of the potential 6′′-*O*-sorboyl-polydatin prodrug in biomass-derived 2-methyltetrahydrofuran (2-MeTHF) was achieved using* Candida antarctica* lipase B for the first time. Under the optimal conditions, the initial reaction rate, maximum substrate conversion, and 6′′-regioselectivity were as high as 8.65 mM/h, 100%, and 100%, respectively. Kinetic and operational stability investigations evidently demonstrated excellent enzyme compatibility of the 2-MeTHF compared to the traditional organic solvents. With respect to the antioxidant properties, three unsaturated ester derivatives showed slightly lower DPPH radical scavenging activities than the parent agent. Interestingly, further studies also revealed that the antiradical capacities of the acylates decreased with the elongation of the unsaturated aliphatic chain length from C4 to C11. The reason might be attributed to the increased steric hindrance derived from the acyl residues in derivatives.

## 1. Introduction

Polydatin (also known as piceid and resveratrol-3-*O*-*β*-mono-D-glucoside) is the key pharmacodynamic constituent from* Polygonum cuspidatum* Sieb. et Zucc. which is an extensively used traditional Chinese medicinal plant in the clinical treatment of platelet aggregation, diabetes mellitus, ischemia–reperfusion injuries, and burn-generated cardiac dysfunction, and so forth [1, 2]. Nowadays, the highly lipophilic drugs are becoming more and more prevalent and well designed with enhanced pharmacological activity in pharmaceutical industry [3, 4]. However, similar to other original natural drugs, polydatin generally suffers from poor solubility, low bioavailability, and unsatisfactory stability in lipid formulations because of its polyhydroxy structure [5]. Consequently, in order to overcome the aforementioned disadvantages and improve the bioavailability in lipid systems, one of the most effective strategies is to synthesize ester prodrugs of polydatin. For example, structure-function relationships revealed that the acylated derivatives of rutin, pruning, and naringin presented improved biological activities and physicochemical properties compared to the corresponding parental agents [6, 7]. Moreover, further investigations also found that natural compounds modified by *α*- or *β*-unsaturated groups usually displayed the higher biological activity compared with the parent agents [8, 9].

Chemically, the acyl chlorides and anhydrides are usually required as acylating reagents for acylating flavonoids, which are characterized by the poor selectivity, tedious protection-unprotection steps, and low product yields [10]. Fortunately, enzymatic strategy instead of conventional chemical method has been well established for acylating compounds possessing similar reactive hydroxyl groups [11]. Recently, our research group has successfully explored an efficient and practical enzymatic route for the regioselective acylation of polyhydroxy nucleosides by using medium engineering, enzyme engineering, and substrate engineering strategies with excellent regioselectivities and conversions [12, 13].

Over the past several years, biobased solvents, that is, green solvents derived from renewable raw materials, have aroused great interest as environmentally friendly alternatives to traditional organic solvents in catalysis and organic chemistry, particularly in biocatalytic reactions with promising physicochemical properties of renewability, biodegradability, environmental benignancy, stability, and so forth [14]. Among these specific solvents, 2-MeTHF produced from renewable resources of lignocellulosic biomass, such as corn crops, bagasse, and oat hulls, exerts clear advantages like the low miscibility with water, high boiling point (82°C), negligible toxicity, higher stability, and abiotic degradability by exposure to sunlight and air [14, 15]. Following the pioneering work of Simeó and coworkers in enzymatic acylation of nucleosides in 2-MeTHF [16], biocatalysts have shown higher activity and stability in this ecofriendly medium than in traditional organic solvents.

**Scheme 1:**
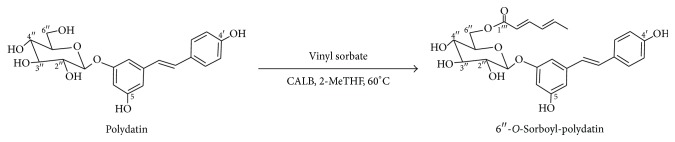
CALB-catalyzed acylation of polydatin with vinyl sorbate.

Therefore, in this study, an effective enzymatic method in 2-MeTHF is explored for preparing potential dual prodrug of sorbic acid (an unsaturated fatty acid with noticeable bactericidal and fungicidal properties) ester of polydatin catalyzed by* Candida antarctica* lipase B (CALB), a versatile enzyme immobilized on macroporous acrylic resin with a molecular weight of 33 kDa and approximate dimensions of 10 Å × 4 Å × 12 Å [17, 18] (Scheme 1). The kinetic study of the acylation was explored in detail to better identify the effect of 2-MeTHF on the performance of the enzyme. Particularly, the free radical scavenging activities of several unsaturated acylated derivatives were also preliminarily studied.

## 2. Materials and Methods

### 2.1. Materials

CALB (*Candida antarctica* lipase B immobilized on macroporous acrylic resin, 10000 U/g) was from Novozymes Co., Ltd., China. Vinyl sorbate, vinyl undecenoate, and vinyl crotonate were obtained from TCI. Polydatin, 2,2-diphenyl-1-picrylhydrazyl (DPPH), 2-MeTHF, and* t*-amyl alcohol were purchased from Sigma-Aldrich. All other reagents were of analytical grade and were dried by 4 Å molecular sieves.

### 2.2. Enzymatic Acylation Procedure

The acylation was conducted with polydatin (0.03 mmol), vinyl sorbate (0.27 mmol), anhydrous solvent (3 mL), and 100 mg lipase while stirring at 200 rpm and at a certain temperature. To determine the concentrations of the polydatin and regioselectivities of the acylated products, aliquot fractions (50 *μ*L) were withdrawn at specified time intervals from the reaction system, followed by a HPLC analysis. All experiments were conducted at least in triplicate and the errors did not exceed 5%. No chemical acylation was detectable as confirmed by the control experiments.

### 2.3. Operational Stability of CALB

After the maximum substrate conversion reached during the batch acylation of polydatin, the immobilized enzyme was separated by filtration and washed three times with 2-MeTHF or THF. Then, the reused prepared catalyst was added into the fresh reaction mixture (3 mL) containing 0.03 mmol polydatin, 0.45 mmol vinyl crotonate, and 100 mg CALB at 60°C and 200 rpm, followed by the assay of its activity and maximum polydatin conversion. The initial activity and maximum conversion obtained in the first batch were set to 100. The residual activity was defined as the ratio of the reused enzyme activity relative to the original enzyme activity in the same reaction system.

### 2.4. Determination of the Enzymatic Kinetic Constants

The kinetic constants of enzymatic sorboylation were investigated at different polydatin concentrations in 2-MeTHF (2–14 mM), THF (5–50 mM), acetone (4–16 mM), and* t*-butanol (2–18 mM). The reactions were carried out in 3 mL solvent containing different polydatin concentration, 9 equiv. of vinyl sorbate, and 100 mg CALB at 60°C and 200 rpm. The kinetic constants (*K*_*m*_ and *V*_max_) were calculated from Hanes-Woolf plots.

### 2.5. Determination of the Enzymatic Apparent Activation Energy (*E*_*a*_)

The apparent activation energy values (*E*_*a*_) of enzymatic acylation in 2-MeTHF, THF, acetone, and* t*-butanol were investigated. Polydatin (0.03 mmol), CALB (100 mg), and vinyl sorbate (0.27 mmol) were added into the anhydrous solvent (3 mL) and incubated at different temperatures (in the range of 35–50°C) and 200 rpm. *E*_*a*_ value of enzymatic sorboylation was calculated according to the linear regression analysis of the Arrhenius plot.

### 2.6. NMR and HPLC Analytical Procedure

Qualitative analysis of the reaction mixtures was conducted by HPLC, using a 4.6 mm × 250 mm (5 *μ*m) Eclipse Plus-C18 column (Agilent Technologies Industries Co., Ltd., USA), a UV detector at 275 nm, and a solvent mixture system of water and methanol (40/60, v/v) at a flow rate of 1.0 mL/min. The retention times for polydatin and 6′′-ester were 2.59 and 5.27 min, respectively. The acylated derivative was purified by flash column chromatography using ethyl acetate (EA)/petroleum ether (PE) and its chemical structure was determined by ^13^C NMR (100 MHz) and ^1^H NMR (400 MHz) (Bruker DRX-400 NMR Spectrometer, Bruker Co., Germany) in DMSO-*d*_6_. 

*6*′′*-O-Sorboyl-polydatin*. ^1^H NMR (DMSO-*d*_6_) *δ*: 9.63 (br s, 1H, OH phenolic), 9.51 (br s, 1H, OH phenolic), 7.43 (d, 2H, *J* = 6.0 Hz, H_2′_ + H_6′_), 7.18–7.13 (m, 1H, *J* = 18.0 Hz, H_4′′′_), 7.05 (d, 1H, *J* = 10.8 Hz, H_vinyl-1_), 6.91 (d, 1H, *J* = 10.8 Hz, H_3′′′_), 6.88 (t, 1H, *J* = 2.4 Hz, H_2′′′_), 6.87 (t, 1H, H_vinyl-2_), 6.81 (d, 1H, *J* = 5.6 Hz, H_3′_), 6.70 (br s, 1H, H_5′_), 6.63 (br s, 1H, H_2_), 6.36 (t, 1H, *J* = 1.6 Hz, H_6_), 6.16–6.12 (m, 1H, H_5′′′_), 5.80 (dd, 1H, *J* = 1.2, 1.2 Hz, H_4_), 5.42 (br s, 1H, OH_2′′_), 5.36 (br s, 1H, OH_3′′_), 5.24 (br s, 1H, OH_4′′_), 4.94 (d, 1H, *J* = 5.2 Hz, H_1′′_), 4.47 (d, 1H, *J* = 7.2, H_6′′-1_), 4.11 (dd, 1H, *J* = 5.2, 5.2 Hz, H_6′′-2_), 3.74–3.71 (m, 1H, H_5′′_), 3.39–3.36 (m, 1H, H_2′′_), 3.32–3.30 (m, 1H, H_3′′_), 3.25–3.22 (m, 1H, H_4′′_), 1.71 (d, 3H, *J* = 4.4 Hz, H_6′′′_). ^13^C NMR (DMSO-*d*_6_) *δ*: 166.77 (C_1′′′_), 159.05 (C_3_), 158.86 (C_5_), 157.85 (C_4′_), 145.64 (C_3′′′_), 140.43 (C_4′′′_), 139.82 (C_1_), 129.86 (C_2′_ + C_6′_), 128.95 (C_1′_), 128.40 (C_vinyl-1_), 125.76 (C_vinyl-2_), 118.75 (C_2′′′_), 116.05 (C_3′_ + C_5′_), 107.62 (C_6_), 104.79 (C_2_), 103.37 (C_1′′_), 100.55 (C_4_), 76.89 (C_2′′_), 74.29 (C_5′′_), 73.62 (C_3′′_), 70.59 (C_4′′_), 64.17 (C_6′′_), 60.24 (C_5′′′_), 18.77 (C_6′′′_).

### 2.7. DPPH Scavenging Activity

The antioxidant activity of polydatin and its acylated derivatives was examined according to the previously reported procedure with a few modifications [19]. Briefly, 2 mL of freshly made DPPH solution was added into ethanol solutions of individual tested compounds to start the reaction at a final concentration of 200 *μ*M DPPH. Each compound concentration was fixed at 0.1, 0.2, 0.4, 0.8, and 1.2 mg/mL, respectively. The absorbance at 517 nm was measured against a blank of pure ethanol in a light proof reaction vessel at room temperature for 30 min. DPPH scavenging capacity was estimated based on the difference in absorbance and expressed by the remaining radical DPPH percentage. Triplicate reactions were carried out for each antioxidant experiment and the results were based on the average values.

## 3. Results and Discussions

### 3.1. Selection of Suitable Reaction Media

The search for suitable solvent capable of maintaining enzyme activity, improving enzyme specificity, and accelerating the enzymatic reaction process has always been a central problem for biochemists [20, 21]. So far, no empirical regularities could be followed to guide the rational choice of the solvent system in nonaqueous enzymology. The solvent physicochemical properties such as the log⁡*P*, dielectric constant, viscosity, dissolving capacity, and molecular size unpredictably affect the behavior of the enzyme. Hence, eight organic solvents with different log⁡*P* values (−1.10–1.43) were selected to further evaluate their effects on the CALB-mediated sorboylation of polydatin.

As shown in Table 1, the initial reaction rate and conversion of the biocatalytic sorboylation varied greatly with the use of different solvents. For the regioselectivity, it is interesting to note that the solvents investigated gave exclusively acylation at the 6′′-hydroxyl group. However, there was not an inevitable correlation between the enzyme activity and solvent properties including the log⁡*P*, viscosity, and substrate solubility in this acylation. In those solvents such as THF, dioxane, cyclohexanone, and* t*-amyl alcohol, CALB displayed poor to good activities with 25.16–79.31% maximal conversions. By contrast, acetone and* t-*butanol seemed to be selectable media and gave 99.00% substrate conversions, but the initial rates were far from satisfactory (0.62–1.64 mM/h). However, when the reaction was carried out in ecofriendly 2-MeTHF, CALB showed highest reaction rate (3.04 mM/h) and excellent conversion (99.57%), indicating that the biomass-derived 2-MeTHF exhibited prominent biocompatibility with the lipase compared to the other conventional organic media. Furthermore, compared with the acetone and* t-*butanol, 2-MeTHF may be more beneficial to maintain enzyme activity owing to its more hydrophobic character [22].

### 3.2. Effect of the Substrate Molar Ratio, Temperature, and Reaction Time

**Figure 1:**
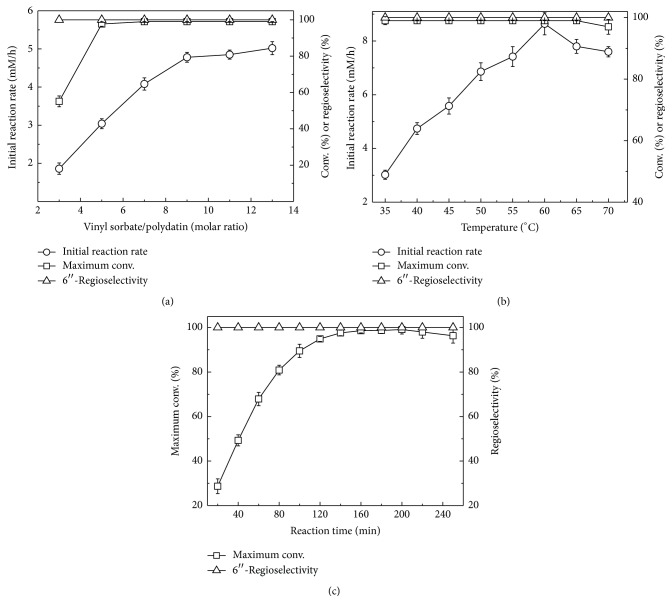
Effect of the molar ratio of vinyl sorbate to polydatin (a), temperature (b), and reaction time (c) on CALB-catalyzed acylation of polydatin. Reaction conditions: (a) 0.03 mmol polydatin, 100 mg CALB, 3 mL anhydrous 2-MeTHF, 200 rpm, various amounts of vinyl sorbate, 40°C; (b) 0.03 mmol polydatin, 100 mg CALB, 3 mL anhydrous 2-MeTHF, 200 rpm, 0.27 mmol vinyl sorbate, different temperatures from 35 to 70°C; (c) 0.03 mmol polydatin, 100 mg CALB, 3 mL anhydrous 2-MeTHF, 200 rpm, 60°C, reaction time from 20 to 250 min.

The influence of some crucial factors, such as the substrate molar ratio, temperature, and reaction time, was also investigated in detail. As can be seen in Figure 1(a), the optimal molar ratio of vinyl sorbate to polydatin was shown to be 9, suggesting that an excessive amount of vinyl sorbate is required due to the reversibility of the reaction. Temperature has a considerable effect on the conformational unfolding of the protein and the reaction equilibrium as well.  Figure 1(b) illustrates that there was 1.9 times increase in the initial rate when the temperature was raised from 35 to 60°C, beyond which further increasing temperature brought a slight drop in the initial reaction rate and maximum conversion. Lipase deactivation caused by high temperature may be one of the reasonable explanations for this phenomenon.

Gardossi et al. recently demonstrated that it is very necessary to monitor the procedure parameters (such as the reaction rate, selectivity, and yield) at a single timepoint [23]. So, the time course of producing 6′′-*O*-sorboyl-polydatin was followed under the optimal conditions for better understanding the enzymatic process. It was found in Figure 1(c) that the rate of substrate conversion underwent a steep increment within 80 min and then a smooth rise with prolonging the incubation time up to 160 min. This phenomenon may occur because the acetaldehyde and sorbic acid from the vinyl sorbate partially inactivated the biocatalyst [24, 25]. After reaching a maximum (200 min), the substrate conversion gradually dropped with extended reaction time, which is in accordance with our previous studies and means that the acylated product was enzymatically hydrolyzed during subsequent reaction process [26].

### 3.3. Operational Stability of the CALB

**Figure 2:**
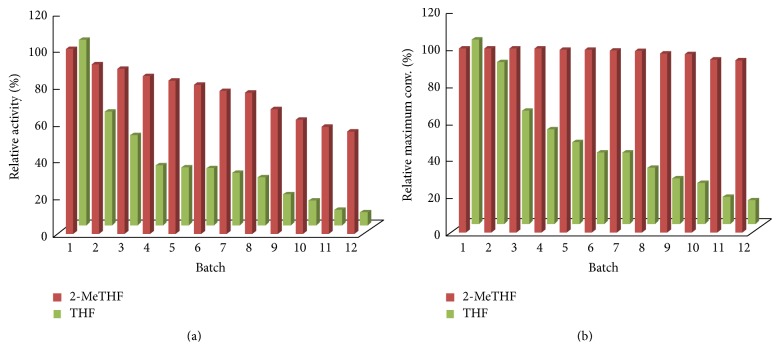
Operational stability of CALB in 2-MeTHF and THF. The reactions were carried out at 60°C and 200 rpm by adding 0.03 mmol polydatin, 0.27 mmol vinyl sorbate, and 100 mg CALB into 3 mL anhydrous 2-MeTHF.

The successful incorporation of biocatalysts into fine chemicals production requires not only high catalytic activities but also excellent operational stabilities. The results for repeated uses of the enzyme in 2-MeTHF and THF are presented in Figure 2 and the differences are evident. The catalytic activity of CALB decreased only slightly during the first two batches in 2-MeTHF, with around 89.27% of its original activity being retained after the second batch, whereas only about 48.73% was obtained in THF. In particular, after eight successive cycles of reuse, CALB still displayed 2.9 times the residual activity (76.34% versus 26.00%) in 2-MeTHF compared to in THF. Similarly, for the maximal conversion of the operational stability, the catalyst remained 93.24% and 13.21%, respectively, of their initial maximal conversions in ecofriendly 2-MeTHF and traditional THF after being used repeatedly for 12 batches, showing that the biomass-derived 2-MeTHF exhibited a great potential as the reaction medium for the enzyme-catalyzed acylation.

### 3.4. Kinetic Studies

For better understanding the superiority of 2-MeTHF, measurement of kinetic constants of enzymatic sorboylation by using linear Hanes-Woolf plots was successfully explored. As illustrated in Table 2, the apparent Michaelis constant *K*_*m*_ value of the enzyme in 2-MeTHF (27.9 mM) is much lower than those attained in* t*-butanol, acetone, and THF (100.3, 103.2, and 107.2 mM, resp.). Simultaneously, the catalytic efficiency of CALB varied greatly, depending on the nature of media. For instance, CALB afforded the highest *V*_max_/*K*_*m*_ value of 1.12 h^−1^ in 2-MeTHF system, which is 3.7- to 5.5-fold higher than that in other solvents studied. These promising observations indicated that the enzyme exerted an extremely higher affinity for the substrate and catalytic efficiency in this novel 2-MeTHF as compared to normal organic solvents.

**Figure 3:**
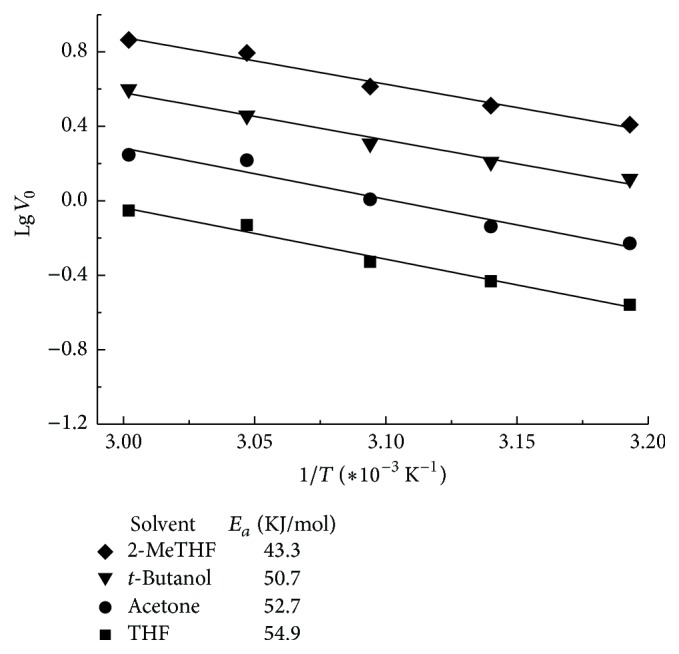
Arrhenius plots of CALB-catalyzed acylation of polydatin in various media. The reactions were carried out at different temperatures and 200 rpm by adding 0.03 mmol polydatin, 0.27 mmol vinyl sorbate, and 100 mg CALB into 3 mL anhydrous 2-MeTHF.

The apparent activation energy (*E*_*a*_) for the reaction is also determined by plotting the data in an Arrhenius plot (Figure 3). The obtained *E*_*a*_ value of 43.3 KJ/mol in 2-MeTHF is lower than that of the sorboylation in other media used (50.7–54.9 KJ/mol) suggesting that the application of 2-MeTHF has proved to be more beneficial for overcoming the reaction energy barrier and rendering the process of enzymatic biotransformation.

### 3.5. Radical DPPH Scavenging Activity

**Figure 4:**
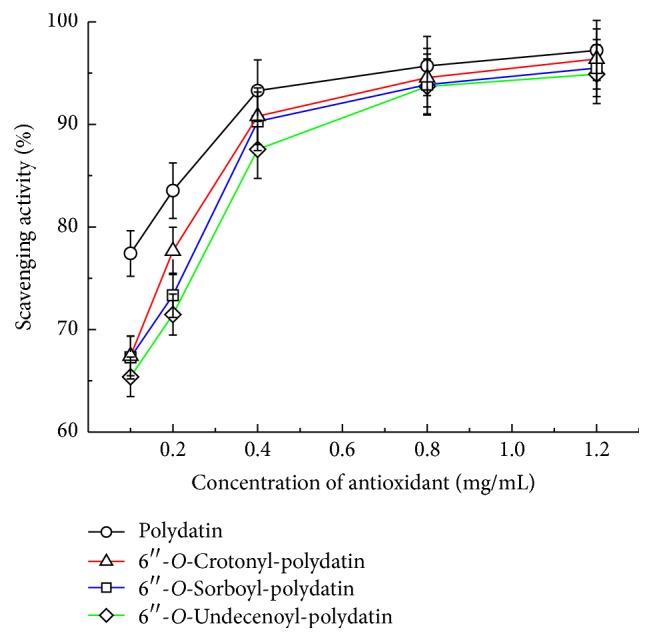
Comparison of radical DPPH scavenging capacity.

Based on the optimization of the enzymatic sorboylation, 6′′-*O*-crotonyl- and 6′′-*O*-undecenoyl-polydatin were also successfully synthesized for better evaluation of the free radical scavenging activity of the unsaturated polydatin ester derivatives. As depicted in Figure 4, all of the tested compounds displayed the obvious dosage-effect relationship on DPPH radical quenching capacity and the higher concentrations were more effective in quenching free radicals. When the concentration was fixed at 1.2 mg/mL, for instance, all the unsaturated acylated products exhibited superior antioxidant activity (94.5–96.4%). However, the scavenging activities of three acylates were always lower than those of the corresponding concentrations of the parent agent. This is in agreement with the previous findings on the acylated flavonoids [27, 28]. For the effect of the aliphatic acid chain length, the unexpected gradual decreases of the free radical scavenging activities were obtained with the increment in the unsaturated aliphatic chain length from C4 to C11. One of the most possible reasons might be that the increased steric hindrance derived from the acyl residues in derivatives prevents the substrate molecule from smoothly entering radical active sites and thus reducing the DPPH scavenging activity.

## 4. Conclusions

In this paper, a practical enzymatic approach for acylating polydatin with vinyl sorbate in biomass-derived 2-MeTHF is described for the first time. CALB lipase was used successfully to acylate polydatin at the 6′′-position. Detailed investigations have revealed that the enzyme in 2-MeTHF gave the lowest *K*_*m*_ and *E*_*a*_ values, highest *V*_max_, and excellent operational stability, which suggested that this ecofriendly solvent could render the enzyme much higher affinity for the substrate and catalytic efficiency. For the antioxidant activity, introducing the acyl group into the polydatin would result in the slight reduction of its DPPH radical scavenging capacity. In addition, more detailed physicochemical investigations on the pH stability, 1-octanol-water partition coefficient (log⁡*P*), apoptosis-inducing capability, and so forth of the acylated products are currently in process and will be reported in due course.

## Supplementary Material

The detailed informations (such as the original HPLC and NMR spectrum) on the sorboyl-polydatin prodrug synthesized by Candida antarctica lipase B in biomass-derived 2-methyltetrahydrofuran are given in the supplementary material.

## Figures and Tables

**Table 1:** Effect of solvent on CALB-catalyzed acylation of polydatin with vinyl sorbate^a^.

Medium	log⁡*P*	Viscosity^b^	Solubility (mM)^c^	*V*_0_ (mM/h)	*C* (%)	6′′-Regioselectivity (%)
2-MeTHF	0.99	0.60	8.51 ± 0.42	3.04 ± 0.15	99.57 ± 0.03	100
*t*-Amyl alcohol	1.24	3.70	5.87 ± 0.21	2.40 ± 0.11	79.31 ± 0.71	100
*t-*Butanol	0.60	3.30	6.54 ± 0.26	1.64 ± 0.05	99.00 ± 0.02	100
Cyclohexanone	1.43	2.20	13.96 ± 0.39	0.83 ± 0.06	66.72 ± 1.01	100
Acetone	−0.23	0.32	12.63 ± 0.53	0.62 ± 0.03	99.00 ± 0.02	100
THF	0.49	0.55	72.17 ± 2.98	0.35 ± 0.01	43.22 ± 1.76	100
Dioxane	−1.10	1.30	20.72 ± 1.01	0.21 ± 0.01	25.16 ± 1.31	100

^a^The reactions were carried out at 40°C and 200 rpm by adding 0.03 mmol polydatin, 0.15 mmol vinyl sorbate, and 100 mg CALB into 3 mL anhydrous solvent.

^b^The viscosity of solvents at 25°C.

^c^The solubility of polydatin was determined by HPLC analyses of the saturated solutions at 25°C.

**Table 2:** Effect of polydatin concentration on enzymatic acylation in various solvents^a^.

Medium	*V*_max_ (mM/h)	*K*_*m*_ (mM)	*V*_max_/*K*_*m*_ (h^−1^)
2-MeTHF	31.2	27.9	1.12
*tert*-Butanol	30.8	100.3	0.41
Acetone	24.7	103.2	0.24
THF	23.3	107.2	0.22

^a^The reactions were carried out at 60°C and 200 rpm by adding various amounts of polydatin, vinyl sorbate (9 equiv.), and 100 mg CALB into 3 mL anhydrous solvent.
