# Supplementary material for: Enzymatic Synthesis of Sorboyl-Polydatin Prodrug in Biomass-Derived 2-Methyltetrahydrofuran and Antiradical Activity of the Unsaturated Acylated Derivatives
Source: Biomed Res Int. 2016 Sep 7;2016:4357052. doi: 10.1155/2016/4357052 (PMC5030401; doi:10.1155/2016/4357052)
Supplement: Supplementary file 1 — The detailed informations (such as the original HPLC and NMR spectrum) on the sorboyl-polydatin prodrug synthesized by Candida antarctica lipase B in biomass-derived 2-methyltetrahydrofuran are given in the supplementary material. [file 4357052.f1.doc]

**Supplementary material**

**Enzymatic synthesis of sorboyl-polydatin prodrug in biomass-derived 2-methyltetrahydrofuran and antiradical activity of the unsaturated acylated derivatives**

**Zhaoyu Wang ^1^****, Yanhong Bi ^1^, Rongling Yang ^1^, Xiangjie Zhao ^1^, Ling Jiang ^2^, Chun Zhu ^1^, Yuping Zhao ^1^, Jianbo Jia ^1^**

*^1^ School of Life Science and Food Engineering,* *Huaiyin Institute of Technology, Huai’an 223003, China*

*^2^ College of Food Science and Light Industry, Nanjing Tech University, Nanjing 211816, China*

Correspondence should be addressed to Zhaoyu Wang; biowzy@126.com and Yanhong Bi; byhfood@126.com

**HPLC analysis**


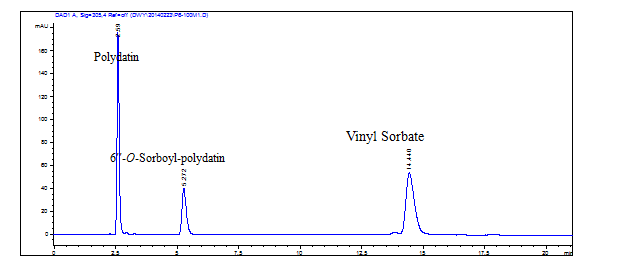


HPLC spectrum of enzymatic sorboylation of polydatin.

**NMR spectrum figures**


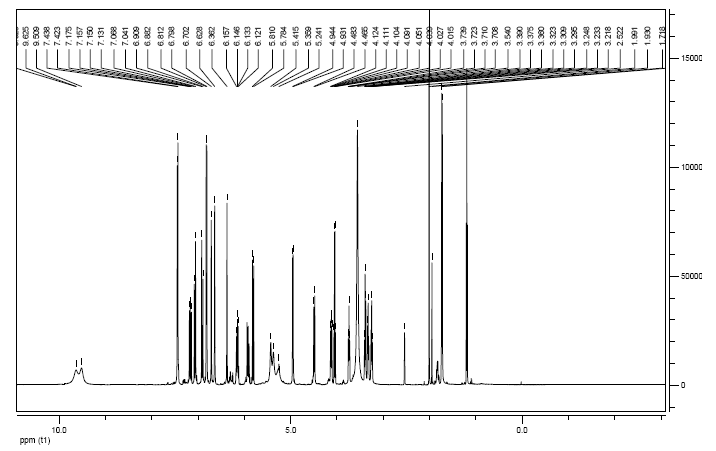


^1^H NMR (6''-*O*-Sorboyl-polydatin in DMSO-*d*_6_)


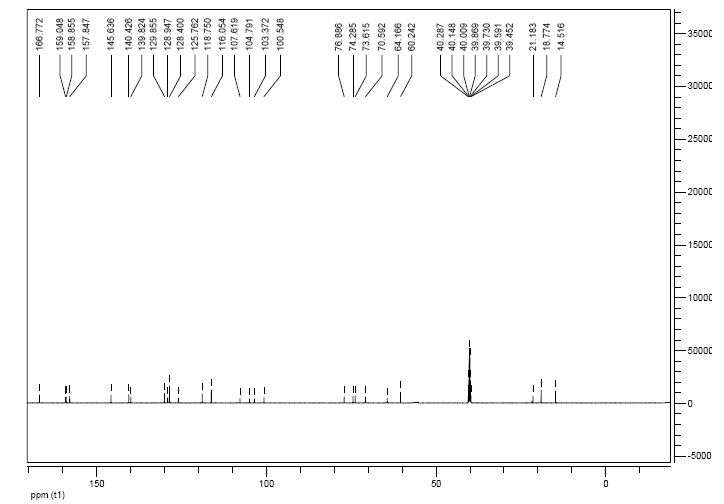


^13^C NMR (6''-*O*-Sorboyl-polydatin in DMSO-*d*_6_)
